# Gene Expression Profiling Unravels Cancer-Related Hepatic Molecular Signatures in Steatohepatitis but Not in Steatosis

**DOI:** 10.1371/journal.pone.0046584

**Published:** 2012-10-10

**Authors:** Julia Starmann, Maria Fälth, Walter Spindelböck, Katja-Lauren Lanz, Carolin Lackner, Kurt Zatloukal, Michael Trauner, Holger Sültmann

**Affiliations:** 1 Unit Cancer Genome Research, German Cancer Research Center and National Center for Tumor Diseases, Heidelberg, Germany; 2 Cellzome/GlaxoSmithKline, Heidelberg, Germany; 3 Laboratory of Experimental and Molecular Hepatology, Division of Gastroenterology and Hepatology, Department of Internal Medicine, Medical University of Graz, Austria; 4 Institute of Pathology, Medical University of Graz, Austria; 5 Division of Gastroenterology and Hepatology, Department of Internal Medicine III, Medical University of Vienna, Vienna, Austria; Institute of Hepatology London, United Kingdom

## Abstract

**Background:**

Pathogenesis and factors for determining progression of alcoholic and non-alcoholic steatosis to steatohepatitis with risk of further progression to liver cirrhosis and cancer are poorly understood. In the present study, we aimed to identify potential molecular signatures for discrimination of steatohepatitis from steatosis.

**Methodology and Results:**

Global microarray gene expression analysis was applied to unravel differentially expressed genes between steatohepatitis compared to steatosis and control samples. For functional annotation as well as the identification of disease-relevant biological processes of the differentially expressed genes the gene ontology (GO) database was used. Selected candidate genes (n = 46) were validated in 87 human liver samples from two sample cohorts by quantitative real-time PCR (qRT-PCR). The GO analysis revealed that genes down-regulated in steatohepatitis were mainly involved in metabolic processes. Genes up-regulated in steatohepatitis samples were associated with cancer progression and proliferation. In surgical liver resection samples, 39 genes and in percutaneous liver biopsies, 30 genes were significantly up-regulated in steatohepatitis. Furthermore, immunohistochemical investigation of human liver tissue revealed a significant increase of AKR1B10 protein expression in steatohepatitis.

**Conclusions:**

The development of steatohepatitis is characterized by distinct molecular changes. The most striking examples in this respect were *KRT23* and *AKR1B10*, which we found to be highly differentially expressed in steatohepatitis compared to steatosis and normal liver. We propose that *KRT23* and *AKR1B10* may serve as future potential biomarkers for steatohepatitis as well as markers for progression to HCC.

## Introduction

Fatty liver diseases comprise a spectrum of severity ranging from simple steatosis over steatohepatitis to cirrhosis and hepatocellular cancer (HCC) [1], [2]. There are two major etiologies for fatty liver disease, namely alcohol and metabolic syndrome-associated disorders such as obesity and type 2 diabetes mellitus (T2DM). Due to its high prevalence and potential for severe hepatic outcomes such as liver cirrhosis and HCC in a substantial fraction of affected individuals, fatty liver disease has become a major issue of public health. Up to 30% of the general population is affected by non-alcoholic fatty liver disease (NAFLD), reaching up to 70% among diabetic patients [3], [4]. The prevalence of steatosis and steatohepatitis in obese patients undergoing bariatric surgery is as high as 76% and 37%, respectively [3], [5]. Steatohepatitis develops in about 20% of alcoholics and up to 50% of T2DM who are also obese (BMI>30). This places fatty liver disease as the most common liver disease of the 21^st^ century accounting for the majority of liver cirrhosis and HCC in Western countries. Its prevalence is expected to further rise in light of the ongoing epidemic of diabetes and obesity [1], [2].

While simple steatosis has a relatively benign course and is principally reversible, steatohepatitis carries a poor prognosis and can lead to severe liver damage with progresson to cirrhosis and HCC. Conventional non-invasive markers such as serum transaminases correlate poorly with the risk of development as well as progression of liver disease, and currently available routine liver tests may even be unremarkable in a significant proportion of patients with steatohepatitis [6]. Therefore in current standard clinical practice, non-invasive serum and imaging markers do not allow the distinction of relatively benign fatty liver from progressive steatohepatitis. This situation results in underdiagnosis and undertreatment of these disorders. The development of efficient diagnostic, prognostic and therapeutic strategies has been substantially hampered by the fact that our understanding of the molecular pathogenesis of steatohepatitis is still incomplete. Several studies showed that the different forms of steatohepatitis (alcoholic – ASH, non-alcoholic – NASH) cannot be morphologically distinguished, which suggests a common pathogenetic mechanism despite different etiologies of the disease [7]. A major unsolved problem is the marked difference in the individual risk to develop steatohepatitis and to progress to cirrhosis (e.g., only 20% of heavy drinkers or 50% of obese type II diabetic patients develop steatohepatitis; Hispanics and Caucasians are more susceptible than Afro-Americans [8], [9]). However, the factors responsible for disease progression across the spectrum of fatty liver disease are poorly understood. Why some patients are protected against developing steatohepatitis or simple steatosis, while others are not, is still unclear [10]. It is currently even debated whether steatosis and steatohepatitis represent two consecutive disease stages; alternatively, individuals may *a priori* be predetermined to develop either a rather benign steatosis or prognostically unfavorable steatohepatitis [4].

In the present study, we performed microarray-based gene expression profiling analysis of steatosis and steatohepatitis and compared them with normal human liver samples. We focused on analyzing transcriptional changes of genes relevant in steatohepatitis but not in steatosis and normal liver to identify potential signatures as basis for further development of biomarkers in the discrimination of steatohepatitis as a prognostically more relevant disease entity. Notably, we aimed at investigating molecular changes between steatohepatitis and steatosis, rather than to differentiate between disease etiologies of NALFD. Overall, we validated the expression of 46 target genes in human liver samples from two cohorts. We demonstrate a hitherto unknown molecular signature for cancer-related changes in steatohepatitis but not in steatosis. Several genes were highly significantly expressed in steatohepatitis compared to steatosis and normal liver. The associated protein of one gene (*AKR1B10*) was expressed in steatohepatitis tissue. The gene signature reported in this study may serve as a valuable tool to distinguish between steatosis and steatohepatitis as well as for future development of diagnostic and prognostic biomarkers. Moreover, the results offer important insights into the pathogenesis of the disease, which may also be relevant for design of future therapeutic strategies.

## Methods

### Patient tissue samples

The detailed clinical, biochemical and histological data of the studied patients are given in table 1, 2 and 3. The study was approved by the ethical review committee at the University of Graz (EK number: 20-119 ex 08/09). The diagnoses of all samples used were histologically validated by a board certified pathologist (C.L.) prior to molecular analysis. For histological analysis, hematoxylin and eosin (H&E) and chromotrope aniline blue (CAB) stained sections were used. A histological diagnosis of steatosis was made if more than 5% of the parenchymal area was occupied by steatosis using routine H&E stain, whereas the diagnosis of steatohepatitis (SH) was based on the presence of hepatocellular ballooning in combination with variable degrees of steatosis and/or inflammation [11], [12]. Stage of liver fibrosis was assessed according to the NASH Clinical Research Network Scoring System for NAFLD [13].

**Table 1:** Clinical, biochemical and histopathological patient characteristics.

Microarray samples				
	Steatohepatitis (n = 8)	Steatosis (n = 14)	Controls (n = 10)	p-value
**Sex (m∶f)**	6∶2	8∶6	5∶5	
**Age (y)**	55 (46–72)	61.5 (37–78)	51 (25–73)	0.37
**BMI (kg/m2)**	25.5 (19.8–39.2)	31 (21.4–30.8)	22.8 (18.3–30.1)	0.17
**ALT (IU/l)**	34 (16–56)	25 (12–359)	20 (5–156)	0.57
**AST (IU/l)**	59 (38–92)	27 (9–398)	22 (10–240)	0.02 a,b
**GGT (IU/l)**	60 (24–804)	46 (14–136)	36 (15–146)	0.26
**AP (IU/l)**	88 (60–261)	88 (42–146)	77 (45–157)	0.55
**Bilirubin (mg/dl)**	3.2 (0.8–7.8)	0.8 (0.3–2.5)	1 (0.2–2)	0.01 a,b
**Cholesterol (mg/dl)**	112 (57–177)	226 (117–270)	174 (44–240)	0.00 a
**Triglycerides (mg/dl)**	97 (57–203)	159 (117–270)	97 (32–172)	0.04 a,c
**Diabetes II (Y/N)**	2/6	3/11	0/10	
**Hyperlipidaemia (Y/N)**	2/6	5/9	0/10	
**Hypertension (Y/N)**	6/2	8/6	0/10	
**Histological steatosis grade (0∶1∶2∶3)**	3*∶1∶4∶0	0∶10∶3∶1	10∶0∶0∶0	
**Lobular inflammation (0∶A1∶A2∶A3)**	1∶3∶3∶1	1∶7∶5∶1	6∶3.1∶0	
**Ballooning (0∶1∶2)**	0∶4∶4	14∶0∶0	10∶0∶0	
**Matteoni (points)**	3 (0–4)	2 (1–2)	n/a	
**Fibrosis (0∶F1∶F2∶F3∶F4)**	0∶0∶0∶2∶6	9∶4∶1∶0∶0	9∶1∶0∶0∶0	
**Apoptosis (0∶1∶2)**	4∶4∶0	7∶5∶2	6∶4∶0	
**Alcoholic/non-alcoholic**	5∶3	0∶14	n/a	

* **…steatosis present, albeit <5% of parenchymal area**.

Samples from the Biobank cohort investigated by microarray analysis. (p-value: a. steatohepatitis vs. steatosis, b. steatohepatitis vs. control, c. steatosis vs. control).

**Table 2:** Clinical, biochemical and histopathological patient characteristics.

qRTPCR biobank samples				
	Steatohepatitis (n = 10)	Steatosis (n = 30)	Controls (n = 18)	p-value
**Sex (m∶f)**	7∶03	14∶16	9∶09	
**Age (y)**	54 (44–72)	64 (37–78)	52.5 (22–73)	0.02 c
**BMI (kg/m2)**	26.1 (19.8–39.2)	25.7 (21–33.5)	23.2 (18.3–30.1)	0.03 c
**ALT (IU/l)**	39 (16–204)	25 (11–359)	21.5 (5–311)	0.33
**AST (IU/l)**	65 (38–441)	28 (9–398)	23.5 (7–240)	0.00 a,b
**GGT (IU/l)**	98 (24–804)	40 (14–286)	35 (9–223)	0.03 a,b
**AP (IU/l)**	132 (60–342)	87 (42–161)	77 (45–157)	0.14
**Bilirubin (mg/dl)**	3.43 (0.8–8.5)	0.72 (0.3–3.3)	0.87 (0.2–2.4)	0.00 a,b
**Cholesterol (mg/dl)**	114 (57–180)	219 (87–301)	160 (44–240)	0.00 a,c
**Triglycerides (mg/dl)**	103 (57–231)	149 (54–712)	95.5 (10–184)	0.02 c
**Diabetes II (Y/N)**	2/8	8/22	1/17	
**Hyperlipidaemia (Y/N)**	3/7	7/23	1/17	
**Hypertension (Y/N)**	3/7	18/12	1/17	
**Histological Steatosis grade (0∶1∶2∶3)**	3*∶1∶5∶1	0∶19∶8∶3	17∶0∶0∶0	
**Lobular inflammation (0∶A1∶A2∶A3)**	2∶3∶3∶2	2∶18∶9∶1	11∶5∶1∶0	
**Ballooning (0∶1∶2)**	0∶5∶5	30∶0∶0	17∶0∶0	
**Matteoni (points)**	4 (0–4)	2 (1–3)	n/a	
**Fibrosis (0∶F1∶F2∶F3∶F4)**	0∶0∶0∶2∶8	23∶6∶1∶0∶0	11∶5∶1∶0∶0	
**Apoptosis (0∶1∶2)**	4∶6∶0	9∶15∶6	12∶5∶0	
**Alcoholic/non-alcoholic**	7∶3	0∶30	n/a	

* **…steatosis present, albeit <5% of parenchymal area**.

Samples from the Biobank cohort analyzed by qRT-PCR (p-value: a. steatohepatitis vs. steatosis, b. steatohepatitis vs. control, c. steatosis vs. control).

**Table 3:** Clinical, biochemical and histopathological patient characteristics.

qRTPCR biopsy samples				
	Steatohepatitis (n = 11)	Steatosis (n = 13)	Controls (n = 5)	p-value
**sex (m∶f)**	6∶05	9∶04	4∶01	
**age (y)**	54 (34–69)	45 (25–62)	43 (28–50)	0.07
**BMI (kg/m2)**	28.8 (25.2–31.6)	28.3 (22.8–35.5)	26.7 (24.3–27.7)	0.19
**ALT (IU/l)**	83 (43–154)	75 (21–253)	66 (39–130)	0.59
**AST (IU/l)**	68 (35–520)	42 (19–137)	41 (33–65)	0.07
**GGT (IU/l)**	426 (98–2195)	171 (17–536)	40 (27–69)	0.00 a, c
**AP (IU/l)**	118 (76–267)	87 (37–224)	55 (38–69)	0.01 b
**Bilirubin (mg/dl)**	1 (0.4–1.7)	0.68 (0.3–3.6)	0.46 (0.4–1.5)	0.27
**Cholesterol (mg/dl)**	258 (131–326)	206 (146–259)	142 (125–177)	0.04 b,c
**Triglycerides (mg/dl)**	185 (127–488)	161 (51–324)	43 (20–56)	0.01 b,c
**Diabetes II (Y/N)**	4/7	3/8	0/5	
**Hyperlipidaemia (Y/N)**	3/8	3/8	0/5	
**Hypertension (Y/N)**	5/6	4/7	0/5	
**Steatosis grade (0∶1∶2∶3)**	0∶3∶3∶4	0∶5∶3∶3	4∶1∶0∶0	
**Lobular inflammation (0∶A1∶A2∶A3)**	0∶2∶5∶3	1∶5∶5∶0	n/a	
**Ballooning (0∶1∶2)**	0∶9∶1	11∶0∶0	5∶0∶0	
**Matteoni (points)**	4 (2–4)	2 (1–2)	n/a	
**Fibrosis (0∶F1∶F2∶F3∶F4)**	0∶5∶1∶0∶4	7∶2∶1∶0∶1	n/a	
**Apoptosis (0∶1∶2)**	4∶6∶0	8∶3∶0	n/a	
**Alcoholic/non-alcoholic**	3∶8	0∶13	n/a	

Samples from the Biopsy cohort used for the validation by qRT-PCR (p-value: a. steatohepatitis vs. steatosis, b. steatohepatitis vs. control, c. steatosis vs. control).

Eighty-seven samples from two independent cohorts of human liver samples with alcoholic and non-alcoholic fatty liver disease were used for this study (table 4). The first cohort (‘Biobank cohort’, n = 58) consisted of non-neoplastic liver tissue samples (controls, i.e. normal liver, n = 18; steatosis, n = 30; steatohepatitis, n = 10, of these 8/2 cirrhotic/non-cirrhotic) obtained from patients who underwent liver resections because of HCC (n = 7), other malignant liver lesions (n = 33, 20/33 colon cancer) or benign liver tumors (n = 7) (table 1 and 2). Eight patients from whom steatosis samples were obtained had received a platin-containing chemotherapy (7/8 FOLFOX) three months prior to resection. Although steatosis can be a side effect of FOLFOX treatment, there was no indication that these cases were related to the chemotherapy. In addition, tissue samples from explanted donor livers not utilized for transplantation (n = 11) were included.

**Table 4:** Overview of samples applied to screening and validation of deregulated genes in steatohepatitis and steatosis.

	Microarray screen	Validation of selected target genes
**Biobank cohort**	N = 32	N = 58
**Biopsy cohort**		N = 29
**total**	**N = 32**	**N = 87**

Sample from the screen were also included in the validation.

The second cohort (‘Biopsy cohort’, n = 29) encompassed percutaneous liver biopsy samples including steatosis (n = 13), steatohepatitis (n = 11, of these, 5/6 cirrhotic/non-cirrhotic), and chronic hepatitis C (CHC, n = 5, all genotype 1) as disease controls with absence of steatosis. In the Biopsy cohort, CHC samples were used as controls since patients with normal liver tissue do not undergo percutaneous liver biopsy (table 3). Informed consent was obtained from all patients prior to use of an aliquot of the liver biopsy tissue for molecular analysis.

### RNA extraction and quality control

Total RNA was isolated from the samples using TRI Reagent® (Molecular Research Center, Cincinnati, OH, USA) according to the manufacturer's protocols. The RNA quality was analyzed using microcapillary electrophoresis (Agilent 2100 Bioanalyzer, Agilent Technologies, Böblingen, Germany). Only samples with RIN (RNA integrity number) of 5.0 or higher were subjected to gene expression array analysis.

### Illumina microarray experiments and data analysis

Global gene expression screenings were performed using Biobank samples only (steatohepatitis n = 8 (6/2 cirrhotic/non-cirrhotic), steatosis n = 14 and controls n = 10). For gene expression analysis, we used whole genome expression microarray Sentrix® Human-6 v3 expression bead chips (Illumina®, San Diego, CA, USA) encompassing 49577 features. The experiments were performed at the Genomics and Proteomics Core Facility of DKFZ Heidelberg using 300 ng/µl RNA and protocols recommended by the supplier.

The raw data was log2-transformed and quantile-normalized. Principal Components Analysis (PCA) was performed to investigate the internal data structure in a way which best explained the variance in the data and to identify potential outliers. The R-package “limma" was used to identify differentially expressed genes [14]. With “limma", a linear model is fitted for each gene and a t-test is used to identify differential expressed genes. P-values were adjusted for multiple testing using the Benjamini-Hochberg (BH) procedure. In addition, a fold change of at least 30% was required to designate a gene as differentially expressed. Resulting data was imported into Ingenuity Pathway analysis (IPA) software (Ingenuity Systems, Redwood City, CA, USA) to identify over- or underrepresented pathways as well as potential biomarkers.

The data discussed in this publication have been deposited in NCBI's Gene Expression Omnibus [15] and are accessible through GEO Series accession number GSE33814 (http://www.ncbi.nlm.nih.gov/geo/query/acc.cgi?token=nrmdhyayykcgavo&acc=GSE33814).

### Quantitative real-time PCR

Quantitative gene expression analysis was performed with 87 human liver tissue samples (Biobank samples: 10 steatohepatitis, 8/2 cirrhotic/non-cirrhotic; 30 steatosis and 18 controls; Biopsy samples: 11 steatohepatitis, 5/6 cirrhotic/non-cirrhotic; 13 steatosis; 5 disease controls from patients with chronic hepatitis C) using real-time PCR (LightCycler®480 Real-Time PCR System, Roche Applied Science, Mannheim, Germany). We applied gene specific primer and probe TaqMan® gene expression assays (Applied Biosystems, Weiterstadt, Germany) and performed relative quantification of all genes. *HPRT1* was used as a house-keeping gene to normalize the data. The Cp value was extracted from the Lightcycler®480 software 1.5.0 and the expression level was calculated with the 2^−ΔCp^ method [16], [17].

### Immunohistochemistry

For immunohistochemical analysis 3 µm thick paraffin sections of liver tissue of chronic hepatitis C, NAFLD associated steatosis (NAFL) and steatohepatitis were dewaxed and rehydrated following standard procedures. For antigen retrieval the sections were microwaved in Target Retrieval Solution, pH 9.0 (Dako REAL ™ S2367; Dako, Glostrup, Denmark), for 40 min at 160 W followed by cooling down for 20 min at RT. The sections were then washed in water and PBS. Blocking was carried out with Dako REAL ™ Blocking Solution for 10 min prior to incubation with antibodies against the aldose reductase AKR1B10 (Novus Biologicals, Littleton, CO, USA) diluted 1∶500 in Dako REAL ™ Antibody Diluent for 60 min at RT. Binding of the antibodies was detected with the Dako REAL ™ EnVision ™ Detection System Peroxidase/DAB^+^, Rabbit/Mouse leading to a reddish-brown reaction product.

AKR1B10 protein expression detected in the cytoplasm of hepatocytes was assessed semiquantitatively as outlined below. The intensity of immunostaining was classified as mild to moderate (score A) or marked (score B), and the amount of positive hepatocytes was estimated by application of numerical scores which were defined as:

Score A or B 0: – AKR1B10 immunostaining in hepatocytes not detected

Score A or B 1: – AKR1B10 positive hepatocytes comprise less than 10% of liver parenchyma

ScoreA or B 2: – AKR1B10 positive hepatocytes comprise between 10–30% of liver parenchyma

Score A or B 3: – AKR1B10 positive hepatocytes comprise more than 30% of liver parenchyma

The AKR1B10 score was then derived from the sum of scores A and B and represents an estimate of the amount and the intensity of immunohistochemically detectable AKR1B10 protein expression in liver parenchyma (table 5).

**Table 5:** Immunohistochemical expression of AKR1B10 in liver parenchyma of patients with steatohepatitis, steatosis and chronic hepatitis C.

Histological diagnosis		Score A*	Score B**	AKR1B10 score (A+B)	M-W-U test
**Steatohepatitis (SH) (n = 9)**	Median	2	2	4	
	Range	1–3	1–5	2–5	
**Steatosis (S) (n = 11)**	Median	1	1	2	SH vs.S: *P = 0.003*
	Range	0–2	0–2	0–4	
**Chronic hepatitis C (CHC) (n = 7)**	Median	1	0	1	S vs. CHC: *P = 0.351* SH vs. CHC: *P = 0.006*
	Range	0–3	0–1	0–4	

* Weak and moderate AKR1B10 expression (% of parenchymal area): 0: no expression; 1: 1–10%; 2:>10–30%; 3:>30%.

** Marked AKR1B10 expression (% of parenchymal area): 0: no expression; 1: 1–10%; 2:>10–30%; 3:>30%.

## Results

### Gene expression pattern clearly separates steatohepatitis from steatosis and controls

To screen for changes in hepatic gene expression distinguishing steatohepatitis from steatosis, liver samples from patients with both alcoholic and nonalcoholic fatty liver disease obtained by surgery (Biobank) were subjected to Illumina gene expression bead chip-based analysis. A total of 32 Biobank samples were investigated (table 1). For the analysis of the microarray data, ASH and NASH cases were grouped together in one “steatohepatitis" group. Importantly, we aimed at investigating the molecular changes between steatohepatitis and steatosis only, not between their etiologies. This appears appropriate since both of the diseases, ASH as well as NASH, are characterized by a greatly overlapping spectrum of morphological changes [18]. Gene expression data were subsequently explored by two data analysis procedures, consisting of (i) identification of differentially expressed genes using limma and (ii) selection of the 1000 genes with the highest variance in the entire data set. In the pair-wise comparisons, we identified 4963 and 2543 genes as significantly (FDR<0.05) differentially expressed between steatohepatitis and controls, and steatohepatitis and steatosis, respectively. The two comparisons, shared 1931 differentially expressed genes. The differences between the gene expression profiles of the normal and steatosis samples are small. The variance within the groups is high and due to that, the power of the statistical test is not sufficient to identify significantly differentially expressed genes between the two groups. The number of differentially expressed genes indicates that steatohepatitis, when compared to normal liver tissue, is characterized by more profound molecular changes than steatosis.

**Figure 1:**
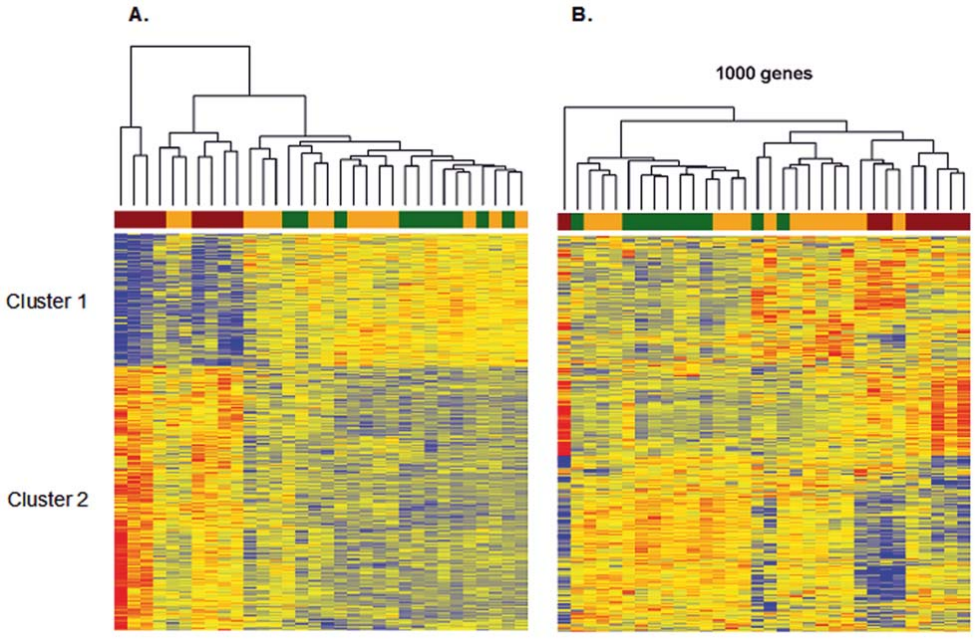
Supervised (A) and unsupervised (B) clustering of differentially expressed genes in the three groups of liver samples. A. Common differentially expressed genes were used for supervised clustering. B. Unsupervised clustering was performed with the 1000 most variable genes in the three groups of liver samples (steatohepatitis, red; steatosis, orange; controls, green).

Hierarchical clustering was used to visualize the 1931 common genes in a heat map (figure 1A). With two exceptions, steatosis and control samples clustered together whereas steatohepatitis samples clustered in a separate branch. Notably, the combination of NASH and ASH did not affect the hierarchical clustering. The common genes were divided into two branches, down-regulated genes in steatohepatitis compared to steatosis and controls (figure 1A, cluster 1) or up-regulated in steatohepatitis compared to steatosis and controls (figure 1A, cluster 2).

The 1000 genes with the highest variance were used for an unsupervised clustering and a visualization of the results in a heat map (figure 1B). Again, with one exception, steatohepatitis samples clustered in a branch separated from steatosis samples and controls. The highly variant genes fell into two classes, characterized by up- and down-regulation in steatohepatitis compared to the remaining samples. Taken together, both types of analyses yielded differential gene expression signatures, which clearly separated steatohepatitis from steatosis and control samples.

Gene ontology [19] analysis was performed for cluster 1 and cluster 2 (table 6 and 7, respectively) to identify disease-relevant biological processes from differentially expressed genes. Notably, cluster 1 (upregulated in controls and steatosis) was characterized by metabolism only (oxidation reduction, metabolic processes, and biosynthesis). In contrast, several GO classes in cluster 2 (upregulated in steatohepatitis) belonged to molecular processes, which are characteristic for malignant diseases and tumor progression (cell adhesion, extracellular matrix, cell motion, integrin signaling). This finding suggests that steatohepatitis is accompanied by entirely different gene expression programs when compared to steatosis and controls. Notably, the programs upregulated in steatohepatitis are characteristic for malignant diseases.

**Table 6:** Gene Ontology (GO) analysis.

	GO.ID	Term	pvalue
**1**	GO:0006805	xenobiotic metabolic process	3.0e-07
2	GO:0055114	oxidation-reduction process	3.7e-07
3	GO:0032787	monocarboxylic acid metabolic process	7.1e-07
4	GO:0006569	tryptophan catabolic process	1.9e-05
5	GO:0042559	pteridine-containing compound biosynthetic process	7.5e-05
6	GO:0009437	carnitine metabolic process	8.9e-05
7	GO:0046874	quinolinate metabolic process	0.0002
8	GO:0070646	protein modification by small protein removal	0.0005
9	GO:0016098	monoterpenoid metabolic process	0.0008
10	GO:0006542	glutamine biosynthetic process	0.0012

Over-represented GO terms for down regulated genes in the common gene list in steatohepatitis samples (Fig. 1A, cluster 1).

**Table 7:** Gene Ontology (GO) analysis.

	GO.ID	Term	pvalue
1	GO:0007155	cell adhesion	1.7e-15
2	GO:0006415	translational termination	1.7e-10
3	GO:0006414	translational elongation	1.8e-10
4	GO:0006935	chemotaxis	2.7e-10
5	GO:0007409	axonogenesis	4.4e-10
6	GO:0030198	extracellular matrix organization	8.4e-09
7	GO:0019083	viral transcription	2.5e-08
8	GO:0016477	cell migration	1.0e-07
9	GO:0031018	endocrine pancreas development	1.0e-07
10	GO:0042060	wound healing	1.1e-06

Over-represented GO terms for up regulated genes in common gene list in steatohepatitis samples (Fig. 1A, cluster 2).

### qRT-PCR validation of microarray data

**Figure 2:**
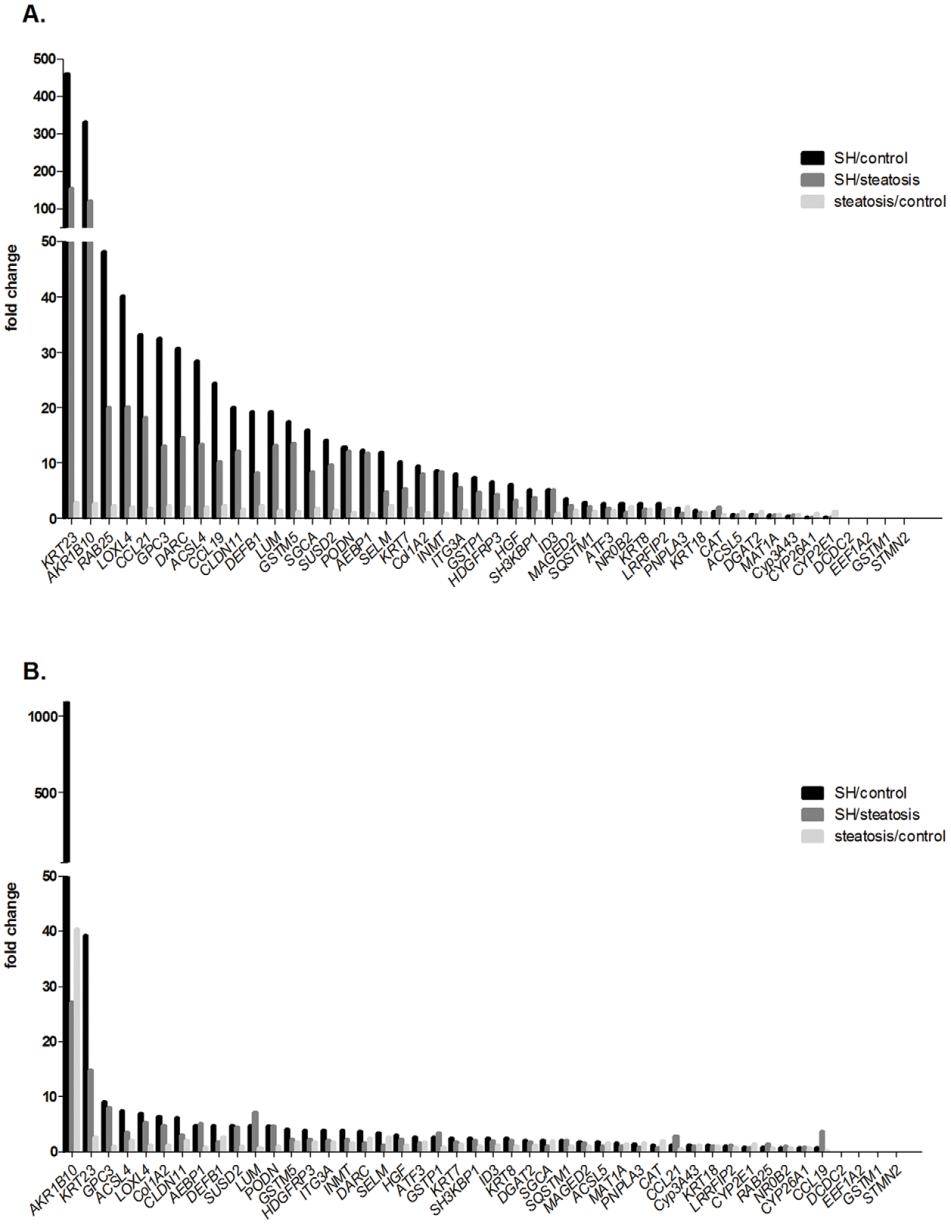
Fold changes of selected genes validated by qRT-PCR. The expression of selected target genes was determined in surgically collected samples (A) and in liver samples obtained by biopsy (B). The fold change was calculated by comparing the three groups of liver samples against each other.

A total of 87 human liver samples were used for PCR-based validation of the array data (table 8 and 9). To optimally select candidate genes for validation, three strategies were employed. First, 265 common genes between the 1931 differentially expressed genes and the 1000 genes with the highest variation were identified. This group of common genes was analyzed by the web-based software IPA® (Ingenuity® systems). IPA-Biomarker® was applied to identify relevant biomarker candidates that could be useful to distinguish between steatohepatitis and steatosis. A total of 126 genes were detected after filtering. From this group, 13 genes were selected for further validation, due to their significant fold change in the microarray analysis. Second, GO terms from the two defined clusters were systematically analyzed for potential targets and three genes were chosen. Third, 17 differentially expressed genes were identified by a significant p-value and fold change from the microarray data. Furthermore, five genes were used as positive controls and additionally, eight genes were chosen due to known function on protein level in steatohepatitis (table 8). Within the selection process of applicable candidates, we mainly focused on genes linked to metabolism, oxidative stress, inflammation, and their relevance in fatty liver disease. In summary, 46 genes were selected for further validation by qRT-PCR. *HPRT1* was used as a housekeeping gene. Figure 2 summarizes the fold changes of the expression of the selected genes. The rates of verification of differential gene expression were high: The changes – as determined by qRT-PCR - of differential expression for 39 genes in Biobank steatohepatitis samples were significant. Only three genes were not significantly deregulated. In biopsies, 30 genes were significant, and twelve genes were not significantly deregulated. The reasons for the lower verification rate in biopsies may be that most of the analyzed Biobank samples had already been used for the whole genome microarray experiment (no independent sample cohort). Four genes (*DCDC2*, *EEF1A2*, *GSTM1* and *STMN2*) could not be reliably measured in any of the samples.

**Table 8:** Candidate genes for qRT-PCR validation.

				Biobank samples					
*Gene*	Reference	Chip/FC (SH/ctrl)	Chip/FC (SH/steatosis)	FC q-RT-PCR (SH/ctrl)	p-value (SH/ctrl)	FC q-RT-PCR (SH/steatosis)	p-value (SH/steatosis)	FC q-RT-PCR (steatosis/ctrl)	p-value (steatosis/ctrl)
***KRT23***	This study	1.261	1.253	460.482	2.29E-10	155.130	9.57E-09	2.968	0.011
***AKR1B10***	IPA biomarker	4.473	4.123	331.812	2.12E-11	121.404	4.29E-11	2.733	0.079
***RAB25***	This study	1.427	1.331	48.235	9.56E-11	20.135	9.44E-10	2.396	0.025
***LOXL4***	This study	3.503	2.564	40.122	7.16E-08	20.196	1.50E-06	1.987	0.018
***CCL21***	IPA biomarker	2.683	2.347	33.277	8.17E-10	18.341	2.13E-08	1.814	0.061
***GPC3***	This study	1.590	1.439	32.522	1.17E-10	13.147	2.02E-08	2.474	3.54E-04
***DARC***	This study	0.804	0.926	30.678	4.88E-07	14.726	1.86E-05	2.083	0.044
***ACSL4***	[20], [21]	1.993	1.825	28.509	1.16E-08	13.526	3.57E-07	2.108	0.029
***CCL19***	IPA biomarker	2.587	2.177	24.562	1.62E-10	10.147	1.75E-08	2.421	0.004
***CLDN11***	IPA biomarker	1.699	1.690	20.043	5.11E-08	12.173	1.88E-06	1.646	0.064
***DEFB1***	This study	2.257	1.661	19.309	4.88E-11	8.200	8.81E-09	2.355	0.001
***LUM***	IPA biomarker	2.509	2.262	19.256	7.43E-08	13.272	2.20E-07	1.451	0.061
***GSTM5***	This study [21]	0.676	0.758	17.483	8.48E-06	13.658	2.18E-05	1.280	0.494
***SGCA***	This study	0.677	0.586	15.864	3.57E-09	8.378	2.06E-07	1.893	0.013
***SUSD2***	IPA biomarker	2.076	1.673	14.207	4.24E-07	9.669	1.16E-06	1.469	0.069
***PODN***	This study	2.046	1.949	12.954	1.72E-08	12.109	1.32E-08	1.070	0.803
***AEBP1***	IPA biomarker	2.277	2.068	12.309	1.26E-07	11.841	6.90E-08	1.040	0.887
***SELM***	This study	1.208	1.224	12.061	5.28E-08	4.938	1.90E-05	2.442	0.002
***KRT7***	This study	1.456	1.083	10.156	3.48E-06	5.372	1.32E-04	1.891	0.026
***COL1A2***	IPA biomarker	1.406	1.588	9.552	2.28E-07	8.076	6.84E-07	1.183	0.482
***INMT***	IPA biomarker	2.038	2.095	8.593	4.03E-06	8.380	5.31E-06	1.025	0.919
***ITGA3***	This study	-	-	8.164	1.99E-07	5.632	2.21E-06	1.450	0.133
***GSTP1***	IPA biomarker	1.457	1.246	7.418	9.03E-07	4.747	1.87E-05	1.563	0.053
***HDGFRP3***	This study	0.157	0.231	6.710	5.51E-06	4.423	9.26E-05	1.517	0.032
***HGF***	This study	0.244	0.260	6.068	3.56E-06	3.324	2.68E-04	1.825	0.002
***SH3KBP1***	This study	0.587	0.539	5.236	9.67E-06	3.789	9.27E-05	1.382	0.143
***ID3***	This study	1.171	1.659	5.206	6.46E-05	5.174	7.44E-05	1.006	0.968
***MAGED2***	This study	0.400	0.389	3.610	1.10E-05	2.476	3.66E-04	1.458	0.010
***SQSTM1***	This study	-	-	2.993	1.50E-04	2.260	0.002	1.324	0.130
***ATF3***	[20]	-	-	2.782	0.001	1.768	0.104	1.573	0.174
***NR0B2***	This study [21]	-	-	2.776	0.008	1.223	0.531	2.270	0.001
***KRT8***	This study	-	-	2.689	0.001	1.628	0.049	1.652	0.041
***LRRFIP2***	This study	−0.398	−0.535	2.665	9.07E-05	1.482	0.037	1.799	0.005
***PNPLA3***	This study	-	-	1.917	0.029	0.894	0.641	2.145	0.002
***KRT18***	This study	-	-	1.434	0.030	1.218	0.209	1.178	0.306
***CAT***	IPA biomarker [20]	−1.389	-	1.406	0.079	1.948	0.197	0.722	0.521
***ACSL5***	This study	−0.896	−0.983	0.780	0.282	0.565	0.026	1.380	0.049
***DGAT2***	This study	-	-	0.715	0.324	0.544	0.074	1.313	0.231
***MAT1A***	IPA biomarker	−1.245	−1.007	0.556	0.012	0.650	0.051	0.855	0.321
***CYP3A43***	This study	−2.113	−1.499	0.435	0.000	0.560	0.060	0.777	0.405
***CYP26A1***	This study	-	-	0.301	0.018	0.288	0.009	1.043	0.916
***CYP2E1***	This study	-	-	0.296	0.021	0.213	0.006	1.388	0.051
***DCDC2***	This study	0.740	0.710	-	-	-	-	-	-
***EEF1A2***	IPA biomarker	3.408	3.158	-	-	-	-	-	-
***GSTM1***	This study	-	-	-	-	-	-	-	-
***STMN2***	IPA biomarker	2.581	2.249	-	-	-	-	-	-

FCs (fold changes) of selected target genes were calculated from gene expression array data for the Biobank cohort. Gene expression from selected target genes was validated by qRT-PCR in the Biobank cohort.. FC was calculated between steatohepatitis (SH) and controls (ctrl) as well as between SH and steatosis.

**Table 9:** Candidate genes for qRT-PCR validation.

	biopsy samples					
*Gene*	FC q-RT-PCR (SH/ctrl)	p-value (SH/ctrl)	FC q-RT-PCR (SH/steatosis)	p-value (SH/steatosis)	FC q-RT-PCR (steatosis/ctrl)	p-value (steatosis/ctrl)
***AKR1B10***	1095.838	4.98E-10	27.173	6.79E-05	40.328	1.23E-05
***KRT23***	39.302	0.001	14.809	0.006	2.654	0.049
***GPC3***	9.060	0.010	8.043	0.004	1.126	0.862
***ACSL4***	7.444	0.001	3.491	0.004	2.132	0.103
***LOXL4***	6.939	1.58E-05	5.274	0.001	1.316	0.449
***Col1A2***	6.362	1.94E-05	4.852	1.67E-04	1.311	0.408
***CLDN11***	6.251	0.001	2.968	0.003	2.106	0.078
***AEBP1***	4.862	3.18E-04	5.130	0.001	0.948	0.894
***DEFB1***	4.794	8.99E-06	1.767	0.066	2.713	0.001
***SUSD2***	4.786	2.50E-04	4.478	2.93E-04	1.069	0.846
***LUM***	4.735	1.54E-04	7.045	4.00E-05	0.672	0.213
***PODN***	4.650	0.001	4.661	4.00E-04	0.998	0.995
***GSTM5***	4.037	0.014	2.291	0.119	1.762	0.283
***HDGFRP3***	3.986	7.93E-05	2.337	0.006	1.706	0.075
***ITG3A***	3.909	0.009	2.170	0.046	1.801	0.221
***INMT***	3.860	0.005	2.389	0.003	1.616	0.208
***DARC***	3.841	1.11E-04	1.591	0.139	2.415	0.010
***SELM***	3.386	2.98E-04	1.240	0.454	2.729	0.005
***HGF***	3.039	2.74E-06	2.291	0.003	1.326	0.215
***ATF3***	2.732	0.005	1.603	0.138	1.704	0.051
***GSTP1***	2.694	0.044	3.364	0.001	0.801	0.599
***KRT7***	2.519	0.014	1.694	0.201	1.487	0.310
***SH3KBP1***	2.472	0.012	2.148	0.003	1.151	0.632
***ID3***	2.456	5.48E-05	1.913	0.013	1.284	0.266
***KRT8***	2.442	0.007	2.146	0.013	1.138	0.637
***DGAT2***	2.207	0.019	1.823	0.041	1.211	0.504
***SGCA***	2.161	0.063	1.138	0.750	1.900	0.125
***SQSTM1***	2.085	0.001	2.089	0.001	0.998	0.991
***MAGED2***	1.821	0.001	1.664	0.025	1.094	0.655
***ACSL5***	1.725	0.016	1.055	0.810	1.635	0.034
***MAT1A***	1.599	0.035	1.098	0.684	1.456	0.046
***PNPLA3***	1.443	0.304	0.932	0.805	1.548	0.177
***CAT***	1.329	0.389	0.682	0.233	1.950	0.003
***CCL21***	1.295	0.539	2.826	0.035	0.458	0.094
***Cyp3A43***	1.216	0.502	1.037	0.906	1.173	0.296
***KRT18***	1.178	0.397	1.101	0.646	1.070	0.719
***LRRFIP2***	1.114	0.723	1.188	0.392	0.938	0.835
***CYP2E1***	0.956	0.875	0.708	0.264	1.351	0.045
***RAB25***	0.916	0.903	1.451	0.616	0.632	0.318
***NR0B2***	0.765	0.557	1.097	0.757	0.698	0.432
***CYP26A1***	0.643	0.348	0.859	0.709	0.749	0.466
***CCL19***	0.634	0.385	3.689	0.032	0.172	0.002
***DCDC2***	-	-	-	-	-	-
***EEF1A2***	-	-	-	-	-	-
***GSTM1***	-	-	-	-	-	-
***STMN2***	-	-	-	-	-	-

Gene expression from selected target genes was validated in the Biopsy cohort. FC was calculated between steatohepatitis (SH) and controls (ctrl) as well as between SH and steatosis.

The genes *ACSL4*, *ATF3*, *CAT*, *GSTM5* and *NR0B2* were previously described in fatty liver disease [20], [21] and served as positive controls for the quantitative validation of candidate genes in the present study. The significant up-regulation of these positive control genes was validated in either one or both of the analyzed cohorts (table 8 and 9).

**Figure 3:**
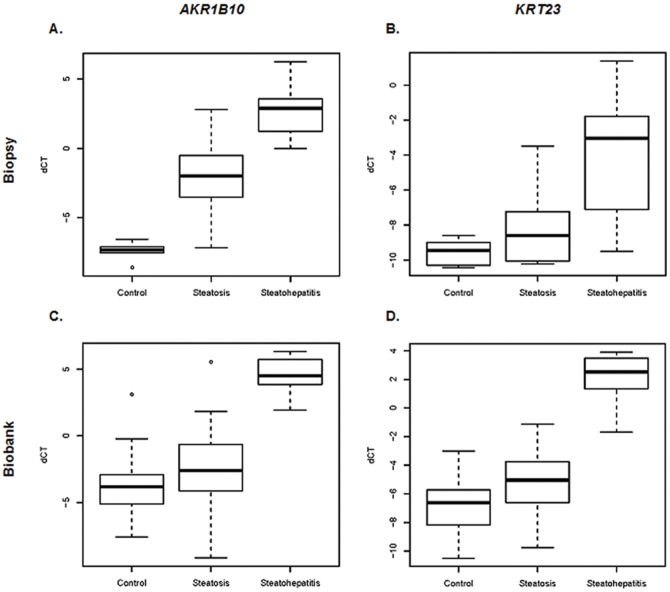
Validation of *AKR1B10* (A and C) and *KRT23* (B and D) mRNA expression by qRT-PCR in biopsy (top) and biobank (bottom) samples. *HPRT1* was chosen to normalize the gene expression in the analyzed samples. Mean normalized expression levels are given in log2.

The major finding supports the notion of a striking alteration of molecular programs in steatohepatitis when compared to steatosis and control samples. The most strikingly deregulated genes in this respect were *AKR1B10* and *KRT23* (figure 3) since our data showed a highly significant overexpression of both genes in steatohepatitis.

### Immunohistochemical analysis of AKR1B10 expression in steatosis, steatohepatitis and chronic hepatitis C

**Figure 4:**
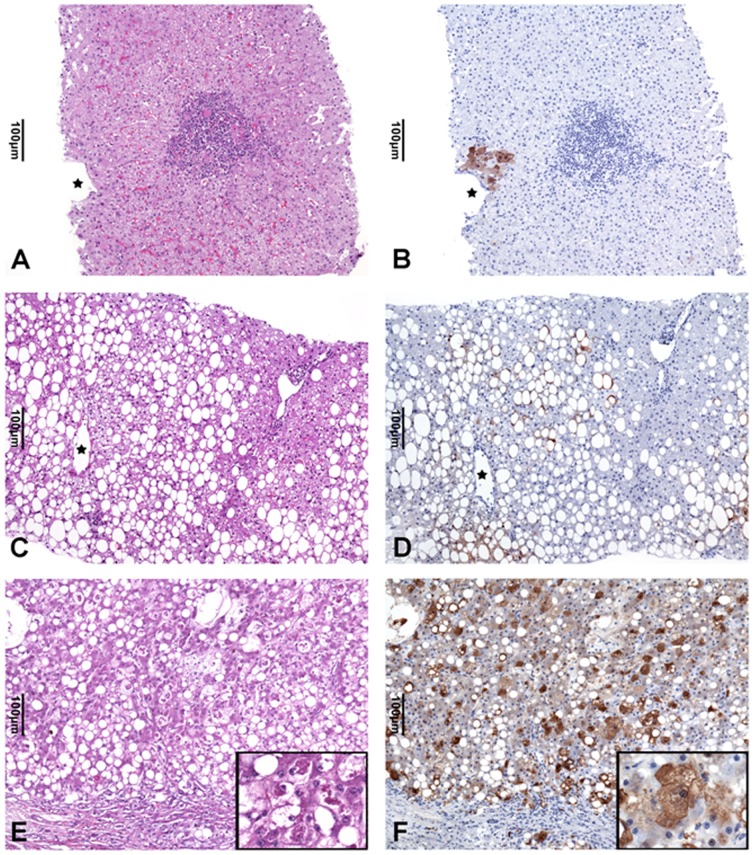
Immunohistochemical detection of the expression of AKR1B10 in chronic hepatitis C (A,B), fatty liver (C,D) and (cirrhotic) steatohepatitis (E,F). Only representative areas are shown. (A) Case of chronic hepatitis C with an inflamed portal tract with lymphocytic infiltrates and mild interphase hepatitis (central vein marked by asterisk, H&E stained section). (B) Consecutive section of the area shown in (A). Only a group of few centrilobular hepatocytes exhibit cytoplasmic and nuclear AKR1B10 immunostaining (central vein marked by asterisk). (C) Case of fatty liver with marked macro- and mediovacuolar steatosis predominantly of centrilobular and mid-zonal hepatocytes (central vein marked by asterisk; H&E stained section). (D) Consecutive section of the area shown in (C) of the hepatocytes with fatty change show staining of the rim of cytoplasm not occupied by fat with AKR1B10 antibodies (central vein marked by asterisk). (E) Case of steatohepatitis in a cirrhotic liver with parenchymal nodule abuting a fibrous septum with mild ductular reaction. Many hepatocytes show fatty change and some of them are characterized by an enlarged, lightly stained cytoplasm (ballooned hepatocytes) and irregular eosinophilic cytoplasmic inclusions (Mallory-Denk bodies, MDBs; inset with higher magnification showing ballooned hepatocytes containing MDBs; H&E stained section). (F) Consecutive section of the area shown in (E). Many of the normal-sized as well as the ballooned hepatocytes show moderate cytoplasmic immunostaining with AKR1B10 antibodies whereas the MDBs remain unstained (inset with higher magnification shows ballooned hepatocytes with MDB).

Weak to moderate or marked cytoplasmic and sometimes nuclear reactivity with antibodies against AKR1B10 was detected in hepatocytes of almost all of the liver tissue samples of patients with steatosis and steatohepatitis of both, alcoholic and non alcoholic etiology, as well as chronic hepatitis C controls (figure 4 A–F, table 5). AKR1B10 expression in cases with steatohepatitis as assessed by the immunohistochemical AKR1B10 score was significantly higher as compared to the AKR1B10 expression in the steatosis and chronic hepatitis C cases (p = 0.003 and 0.006, respectively). However, AKR1B10 expression in liver tissues with steatosis or chronic hepatitis C was not different (p = 0.351) (Table 5). AKR1B10 expression was also detected in the epithelium of some of the large or medium sized bile ducts (data not shown).

## Discussion

The present study unravels gene expression signatures profoundly distinguishing steatohepatitis from steatosis and normal liver. Notably, normal tissue and steatosis clustered more closely together when compared to the steatohepatitis samples. Since the hierarchical clustering clearly demonstrated common expression profiles for NASH and ASH samples, no further distinction was made between the different etiologies. Furthermore, both etiologies manifest with a broadly overlapping spectrum of histological key features (e.g. centrilobular based features of steatosis, inflammation and hepatocellular ballooning as well as pericellular fibrosis) [7], [18].

The gene expression data suggests that steatohepatitis exhibits molecular profiles which are characteristic for processes relevant in malignant tumors and may therefore reflect a premalignant state of liver disease already at the precirrhotic stage (table 7.). Indeed, increasing evidence supports the fact that steatohepatitis can progress to HCC already in precirrhotic stage [22]. Obesity and diabetes are not only established risk factors for developing steatohepatitis and cirrhosis, but have also been implicated in the formation of HCC. Other major risk factors for HCC in steatohepatitis are advanced age and tissue fibrosis. The mechanisms underlying the progression from steatohepatitis to HCC are still unclear, and targets for the treatment of steatohepatitis and HCC are missing. However, signaling pathways involved in inflammation and insulin resistance promoting steatohepatitis, may contribute to its carcinogenic potential.

The gene expression of the 46 selected genes was validated by qRT-PCR for both of the tested sample cohorts. Although the analyzed samples were collected by different approaches, and for the biopsy samples chronic hepatitis C samples were used as controls (since percutaneous liver biopsy is not performed in individuals with normal liver), many significantly deregulated genes were found in all three tested groups of liver samples in both independent sample cohorts (table 1 to 3). Despite a difference in the percentage of cirrhotic cases between the two cohorts (80% in surgical samples, and 45% in biopsies), *AKR1B10* as well as *KRT23* were significantly high expressed in both sample cohorts. This indicates common pathogenetic mechanisms in both alcoholic and non-alcoholic steatohepatitis. Among the differentially expressed genes, *AKR1B10* and *KRT23* were the most prominent ones. AKR1B10 belongs to the aldose reductases and was first described in human HCC [23], [24]. It is a monomeric enzyme reducing aldehydes and ketones to the corresponding alcohols. The protein is expressed in cervical cancer and non-small cell lung cancer, where it is considered as a potential diagnostic biomarker [25], [26], [27]. Several findings also support the hypothesis that *AKR1B10* may be a useful marker for differentiation and proliferation of liver, colon, lung and breast cancer [28], [29], [30]. In addition, AKR1B10 protein plays a role in the detoxification of toxic aldehydes. Free radicals generated by reactive oxygen species oxidize fatty acids of the lipid membrane resulting in the production of reactive aldehydes, which are rapidly reduced by AKR1B10 thereby protecting cells from toxification [31], [32]. Lipotoxicity of fatty acid and lipid intermediary metabolites may be a key event in the progression of fatty liver disease by inducing hepatocellular death. In the present study, we report the significant overexpression of *AKR1B10* in steatohepatitis which could be caused by the increased need to inactivate toxic components in hepatocytes. This suggests that *AKR1B10* may be a molecular marker accompanying the progression of steatohepatitis to HCC.

Genes involved in lipid partitioning by keeping potentially lipotoxic fatty acids stored in neutral triglycerides (TG) may counteract/protect from lipotoxicity as a potentially important factor in progression of NAFLD to steatohepatitis [33]. Notably, some of the genes which were found to be deregulated included enzymes involved in FA homeostasis such as DGAT2 (responsible for FA esterification to TGs) and PNPLA3 (adiponutrin), recently identified as a key determinant for pathogenesis and progression of alcoholic and non-alcoholic fatty liver disease [34], [35].

Furthermore, we describe the highly significant overexpression of *KRT23* in steatohepatitis compared to steatosis and normal liver. *KRT23* has been detected in different cancer types. In microsatellite stable colon cancer, *KRT23* expression is highly increased, and this may have a protective function counteracting the proliferation and survival of cells [36]. Another study identified KRT23 as a HCC-associated antigen in patient sera [37]. *KRT23* has not yet been described to be expressed in liver or liver disease. The role of KRT23 under physiological conditions and in steatohepatitis is unknown. However, mutations of other members of the keratin multigene family, namely keratins 8 and 18, were found to be associated with chronic human liver disease. Furthermore, disturbances of the expression levels of keratins 8 and 18 led to formation of Mallory-Denk bodies, an important morphological characteristic of steatohepatitis [38], [39]. Nevertheless, the functional role of *KRT23* in liver or liver diseases needs to be resolved.

In summary, we propose that *AKR1B10* and *KRT23* may represent pre-malignant markers in the progression of steatohepatitis to HCC. These marker genes can be useful to test patients for a potential development of HCC and for therapeutic decisions in clinical practice. However, further studies will be needed to address the use of these signatures in predicting prognosis and disease course in patients with fatty liver disease.
